# The association between helicobacter pylori infection and Triglyceride-Glucose (TyG) index in US adults: A retrospective cross-sectional study

**DOI:** 10.1371/journal.pone.0295888

**Published:** 2025-01-03

**Authors:** Wei Fu, Junlong Zhao, GuoBin Chen, Linya Lyu, Yao Ding, Liang-Bi Xu

**Affiliations:** 1 Department of Gastroenterology, 925th Hospital of PLA Joint Logistics Support Force, Guiyang, China; 2 Department of Medical Genetics and Developmental Biology, State Key Laboratory of Cancer Biology, School of Basic Medicine, Fourth Military Medical University, Xi’an, China; 3 Department of the Digestive Endoscopy, The Affiliated Hospital of Guizhou Medical University, Guiyang, China; Università degli Studi di Milano, ITALY

## Abstract

**Background:**

The Triglyceride-glucose (TyG) index is a marker for insulin resistance and metabolic syndrome, while Helicobacter pylori is linked to gastrointestinal diseases and may affect metabolic risks. This study examined the association between the TyG index and H. pylori infection in adults.

**Methods:**

Data from 3797 participants in the NHANES 1999–2000 cycle were analyzed. The relationship between the TyG index and H. pylori infection was assessed using multivariate logistic regression and a two-piecewise logistic model to explore non-linear effects. Subgroup analyses were conducted based on age, sex, glucose levels, BMI, and CKD.

**Results:**

A linear association between the TyG index and H. pylori infection was found. Subgroup analyses revealed significant interactions with a few variables.

**Conclusions:**

This study indicates a linear relationship between the TyG index and H. pylori infection, suggesting metabolic influences on H. pylori infection and potential for targeted interventions in at-risk groups.

## Introduction

The Triglyceride-glucose (TyG) index is a proxy for insulin resistance and is associated with metabolic syndrome and cardiovascular diseases [1–3]. Insulin resistance may influence immune responses, potentially affecting susceptibility to infections like those caused by Helicobacter pylori [4–6]. H. pylori, a prevalent gastric pathogen, is linked to gastritis, peptic ulcers, and gastric cancer [7–9], and has been associated with cardiovascular and metabolic disturbances [10–12]. The role of the TyG index in the context of H. pylori infection remains unclear.

While some studies suggest H. pylori infection may influence glucose metabolism and insulin resistance [13, 14], the evidence is inconsistent. A meta-analysis indicates a potential association between H. pylori infection and metabolic syndrome and insulin resistance [15], but the specific relationship with the TyG index has not been explored.

Our study novelly examines the association between the TyG index and H. pylori infection in a large adult population. By employing linear and non-linear models and conducting subgroup analyses, we aim to provide insights into metabolic factors affecting H. pylori infection, potentially informing management and prevention strategies. This research addresses a gap in understanding the metabolic influences on H. pylori infection and the potential utility of the TyG index in at-risk populations.

## Methods

### Study population

The National Center for Health Statistics (NCHS) is responsible for conducting the National Health and Nutrition Examination Survey (NHANES), which is an ongoing research project supported by the Centers for Disease Control and Prevention (CDC) [16]. This study has adopted a composite sampling technique, including multi-stage, stratified, and cluster probability sampling methods, to ensure that the sample adequately represents the U.S. population [17–19]. The study data is derived from the 1999–2000 NHANES. Participants in this cycle included those with data on Helicobacter pylori infection and Tyg index [20, 21]. All procedures received approval from the CDC Ethics Review Board, and written informed consent was secured from all participants. Since the investigators had no access to identifying information, the data analyzed in this study were anonymized and publicly available on the NHANES website. Consequently, the 925th Hospital Review Board determined that this study qualified as "non-human subjects" research [20].

The inclusion criteria for this study were based on the NHANES 1999–2000 cycle, with a total of 9965 subjects participating. Exclusion criteria were applied, which included participants with missing data for H. pylori serology, participants with missing data for gastric diseases, and participants with missing data for covariates.

### Variables

The primary independent variable of interest in this study is the baseline measurement of the triglyceride-glucose (TyG) index. The triglyceride-glucose (TyG) index was calculated using the formula: TyG = ln[(fasting triglycerides (mg/dL) × fasting glucose (mg/dL)) / 2], where "ln" denotes the natural logarithm [21]. The dependent variable is the presence of Helicobacter pylori infection, which is assessed using a dichotomous variable. In this study, standard ELISA thresholds were employed to classify participants as seropositive for Helicobacter pylori (optical density (OD) value ≥1.1) or seronegative (OD value <0.9). Equivocal OD values ranging from 0.9 to 1.1 were excluded to avoid the potential for confounding statistical results [22, 23].

The following variables were included in the fully-adjusted model based on the following criteria: demographic data, variables reported in previous literature to affect the TyG index or Helicobacter pylori, and variables based on clinical experiences. The fully-adjusted model included the following variables: Continuous variables obtained at baseline: age, serum C-reactive protein, Glucose, and Triglycerides. Categorical variables obtained at baseline: gender, education, race, high blood pressure, own housing, alcohol behavior, smoke behavior, BMI, gastrointestinal illness, and CKD(Chronic kidney disease) [24–30]. Patients with Chronic Kidney Disease (CKD) were defined as those with an estimated Glomerular Filtration Rate (eGFR) of less than 60 ml/min/1.73 m2 and/or proteinuria (dipstick results positive at or above 1+), demonstrating stable kidney function for at least three months prior to the study commencement [31], The selection of covariates in our study was guided by the criteria and methodologies outlined in relevant literature.

### Statistical analysis

Categorical variables were expressed as frequencies and percentages. The differences among the TyG groups (quartiles) were analyzed using various statistical tests: the χ2 test for categorical variables, the t-test for normally distributed variables, and the Mann-Whitney U test for variables with skewed distributions [32].

The analysis was conducted in three stages. The first stage involved the application of multivariate binary logistic regression models, resulting in three distinct models: Model 1 without any covariate adjustments; Model 2 adjusted solely for sociodemographic data; and Model 3, which incorporated the covariates listed in Table 1. The final stage entailed subgroup analyses using stratified binary logistic regression models. Continuous variables were categorized based on clinical thresholds or tertiles prior to conducting an interaction test, and effect modification was assessed through the likelihood ratio test.

**Table 1:** Baseline characteristics of the study population: Comparison between the helicobacter pylori seropositive and seronegative groups.

Variables	Total (n = 3557)	HP seronegative (n = 2005)	HP seropositive (n = 1552)	P value	statistic
AGE, Mean ± SD	49.5 ± 18.5	47.0 ± 18.7	52.8 ± 17.6	< 0.001	88.631
Gender, n (%)				0.072	3.232
Male	1697 (47.7)	930 (46.4)	767 (49.4)		
Female	1860 (52.3)	1075 (53.6)	785 (50.6)		
Education (%)				< 0.001	292.905
Less than high school	619 (17.4)	157 (7.8)	462 (29.8)		
High school above	2938 (82.6)	1848 (92.2)	1090 (70.2)		
Race, n (%)				< 0.001	519.459
Mexican	1671 (47.0)	1271 (63.4)	400 (25.8)		
Other Hispanic	603 (17.0)	268 (13.4)	335 (21.6)		
non-hispanic white	961 (27.0)	323 (16.1)	638 (41.1)		
Non-Hispanic black	322 (9.1)	143 (7.1)	179 (11.5)		
High blood pressure (%)				< 0.001	48.994
Normal	1368 (38.5)	858 (42.8)	510 (32.9)		
pre-Hypertensive	1299 (36.5)	721 (36)	578 (37.2)		
hypertension	890 (25.0)	426 (21.2)	464 (29.9)		
own.housing, n (%)				< 0.001	40.596
No	735 (20.7)	338 (16.9)	397 (25.6)		
Yes	2822 (79.3)	1667 (83.1)	1155 (74.4)		
Alcohol behavior, n (%)				0.007	7.197
No	1166 (32.8)	620 (30.9)	546 (35.2)		
Yes	2391 (67.2)	1385 (69.1)	1006 (64.8)		
Smoke behavior, n (%)				0.016	8.268
Never	1854 (52.1)	1084 (54.1)	770 (49.6)		
Sometimes	739 (20.8)	388 (19.4)	351 (22.6)		
Everyday	964 (27.1)	533 (26.6)	431 (27.8)		
BMI.cut, n (%)				< 0.001	11.326
<25	1126 (31.7)	681 (34)	445 (28.7)		
> = 25	2431 (68.3)	1324 (66)	1107 (71.3)		
Gastric illness, n (%)				0.679	0.172
No	3228 (90.8)	1816 (90.6)	1412 (91)		
Yes	329 (9.2)	189 (9.4)	140 (9)		
CRP,(mg/dL)	0.2 (0.1, 0.6)	0.2 (0.1, 0.6)	0.3 (0.1, 0.6)	< 0.001	13.433
CKD, n (%)				0.01	6.598
No	128 (3.6)	58 (2.9)	70 (4.5)		
Yes	3429 (96.4)	1947 (97.1)	1482 (95.5)		
Glucose, (mmol/L)	5.4 ± 2.0	5.2 ± 1.7	5.6 ± 2.3	< 0.001	34.907
Triglycerides, (mmol/L)	1.3 (0.9, 2.0)	1.3 (0.9, 1.9)	1.4 (1.0, 2.1)	< 0.001	22.559
TyG., Mean ± SD	5.8 ± 0.7	5.7 ± 0.6	5.8 ± 0.7	< 0.001	43.521

Other race/ethnicity includes all race/ethnicity other than Mexico-American, non-Hispanic white and black. CRP, C reactive protein; BMI, Body mass index. CKD, chronic kidney disease. T value is for continuous variables and X2 Value is for categorical variables. Bold represents statistically significant. Mean ± SD

To confirm the robustness of the data analysis, a sensitivity analysis was performed. The TyG index was transformed into a categorical variable, and a P-value for trend was computed. The purpose of this analysis was to corroborate the findings obtained when the TyG index was treated as a continuous variable and to investigate potential nonlinearity.

Statistical analyses were conducted using the R software package version 4.2.2 (The R Foundation, http://www.R-project.org) and Free Statistics software version 1.9. A two-tailed test was employed, with a p-value less than 0.05 deemed indicative of statistical significance.

## Results

### Baseline characteristics of selected participants

**Fig 1:**
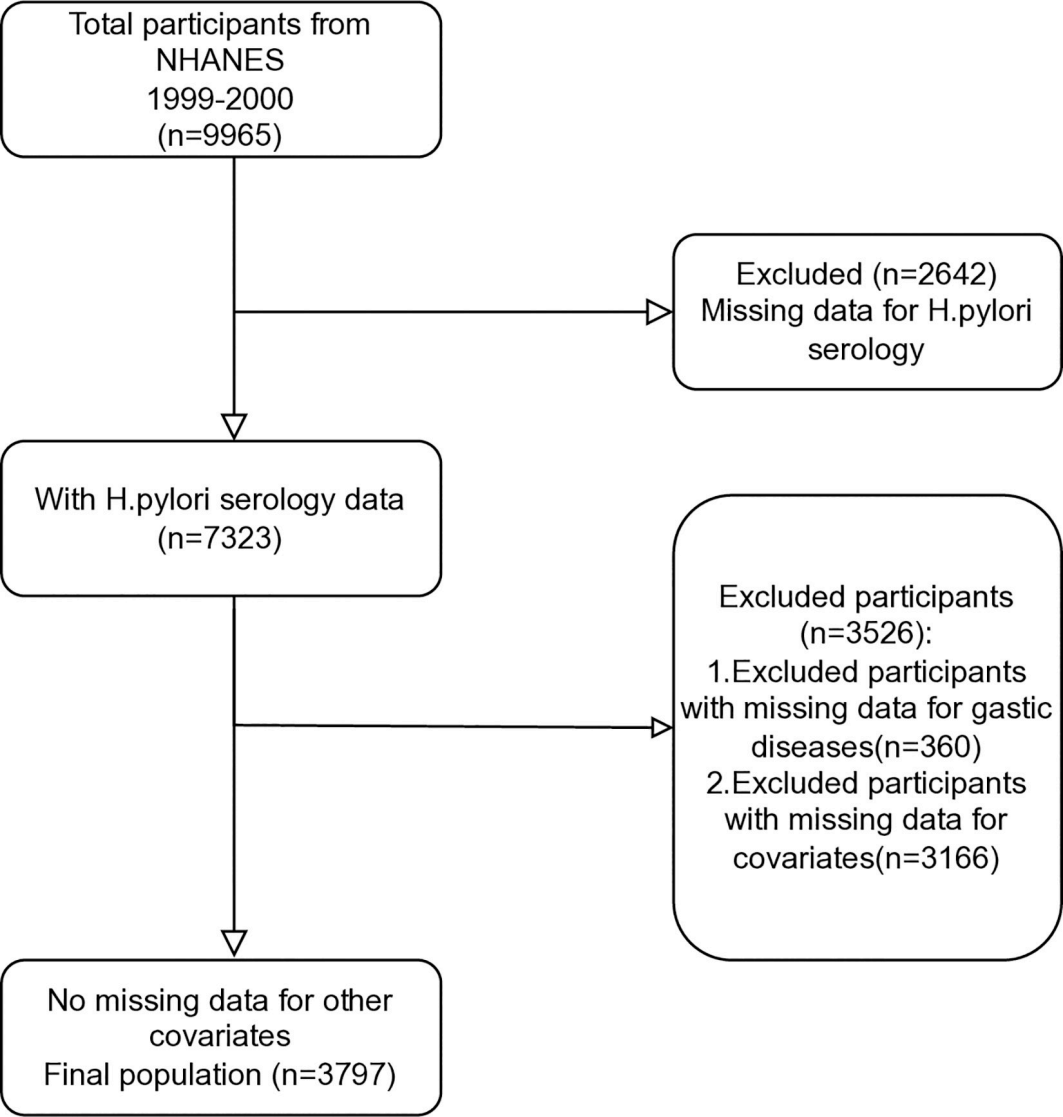
Flowchart for the selection of study participants from NHANES 1999–2000.

Total of 3797 participants were included in the final data analysis, as illustrated in Fig 1 (please refer to the flow chart). The baseline characteristics of the selected participants, stratified by the quartiles of the TyG index, are presented in Table 1. The study cohort had an average age of 41.8 years, with a majority being of Mexican descent (47%) and having an education level higher than high school (82.6%). Among the participants, 32.8% had never smoked, and 52.1% did not drink alcohol. The mean TyG index for the cohort was 5.8.

A notable finding was that participants without Helicobacter pylori (HP) infection had a significantly lower average TyG index, were younger, and had a lower BMI compared to those with HP infection. This suggests a potential association between higher metabolic risk, as indicated by the TyG index, and increased susceptibility to HP infection.

In terms of comorbidities, 25% of the study cohort reported having hypertension. The group with HP infection exhibited a higher incidence of hypertension and a history of cardiovascular diseases compared to the group without HP infection. These baseline characteristics provide valuable insights into the demographic and health-related factors that may influence the risk of HP infection and its potential metabolic correlates.

### Univariate analysis

Table 2 summarizes the univariate analysis of risk factors associated with Helicobacter pylori infection, reporting the disease risk in terms of OR and 95% CI. age, education, race, own housing, high blood pressure, and TyG were significantly associated with Helicobacter pylori infection (p<0.001). Other factors, including gastric illness and CRP, showed no significant association with Helicobacter pylori infection (Table 2).

**Table 2:** Univariate analysis of risk factor associated with Helicobacter pylori seropositivity.

Variable	OR(95%CI)	P-value
Age	1.02 (1.01~1.02)	<0.001
Sex		
Male	Ref.	
Female	0.89 (0.78~1.01)	0.072
Education		
Less than high school	Ref.	
High school above	0.2 (0.16~0.24)	<0.001
Race, n (%)		
Mexican	Ref.	
Other Hispanic	3.97 (3.26~4.83)	<0.001
non-hispanic white	6.28 (5.27~7.47)	<0.001
Non-Hispanic black	3.98 (3.11~5.09)	<0.001
own.housing		
No	Ref.	
Yes	1.71 (1.45~2.01)	<0.001
Alcohol behavior		
No	Ref.	
Yes	1.21 (1.05~1.4)	0.007
Smoke behavior, n (%)		
Never	Ref.	
Sometimes	1.27 (1.07~1.51)	0.006
Everyday	1.14 (0.97~1.33)	0.106
BMI, n (%)		
<25	Ref.	
> = 25	1.28 (1.11~1.48)	0.001
Gastric illness, n (%)		
No	Ref.	
Yes	1.05 (0.83~1.32)	0.679
High blood pressure (%)		
Normal	Ref.	
pre-Hypertensive	1.35 (1.16~1.57)	<0.001
Hypertension	1.83 (1.54~2.17)	<0.001
CKD, n (%)		
No	Ref.	
Yes	0.63 (0.44~0.9)	0.011
CRP	1.06 (0.99~1.14)	0.115
TyG	1.4 (1.26~1.55)	<0.001

To assess the independent effect of the TyG index on Helicobacter pylori, we developed three univariate and multivariate binary logistic regression models. Table 3 illustrates the odds ratio (OR) as the effect size and the corresponding 95% confidence intervals (CIs). In the unadjusted model (Model 0), the effect size represents the change in the risk of Helicobacter pylori infection associated with a one-unit difference in the TyG index. For instance, in the unadjusted model, a one-unit difference in the TyG index implies a 40% increase in the risk of Helicobacter pylori infection, with a 95% CI of [1.26, 1.55]. In the minimally adjusted model (Model 1), a one-unit increase in the TyG index is linked to a 27% rise in the risk of Helicobacter pylori infection, with a 95% CI of [1.14, 1.41]. In the fully adjusted model (Model 3), encompassing all covariates as displayed in Table 1, a one-unit increase in the TyG index is associated with an 18% increase in the risk of Helicobacter pylori infection, with a 95% CI of [1.04, 1.34].

**Table 3:** Multivariate logistical regression for TyG on Helicobacter pylori seropositivity.

	Model 0		Model1		Model2		Model3	
Variable	OR(95% CI)	P value	OR(95% CI)	P value	OR(95% CI)	P value	OR(95% CI)	P value
TyG	1.4 (1.26~1.55)	<0.001	1.27 (1.14~1.41)	<0.001	1.17 (1.04~1.31)	0.01	1.18 (1.04~1.34)	0.008
Q1(<5.203)	1(Ref)		1(Ref)		1(Ref)		1(Ref)	
Q2(> = 5.203,<5.553)	1.15 (0.93~1.42)	0.196	1 (0.81~1.25)	0.986	0.97 (0.76~1.23)	0.797	0.97 (0.76~1.24)	0.824
Q3(> = 5.553,<5.86)	1.21 (0.98~1.49)	0.083	1.04 (0.84~1.29)	0.723	0.93 (0.73~1.18)	0.533	0.93 (0.73~1.19)	0.577
Q4(> = 5.86,<6.249)	1.41 (1.14~1.75)	0.001	1.2 (0.96~1.49)	0.108	1.15 (0.9~1.47)	0.257	1.16 (0.9~1.49)	0.243
Q5(< = 6.249)	1.91 (1.55~2.36)	<0.001	1.54 (1.23~1.91)	<0.001	1.31 (1.03~1.68)	0.031	1.33 (1.03~1.73)	0.028
P for trend		<0.001		<0.001		0.01		0.009

Model 0: No covariates were adjusted.

Model 1: Adjusted for Age, Gender.

Model 2: Adjusted for Age, Gender, Edu, Race, Own.housing, Alcohol, Smoke.

Model 3: Adjusted for Age, Gender, Edu, Race, Own.housing, Alcohol, Smoke, BMI, Gastric. Illness, HBP, CKD, CRP.

### Sensitivity analysis

For the sensitivity analysis, the TyG index was converted from a continuous variable to a categorical variable (five groups of the TyG index). The P-value for trend of the TyG index with categorical variables in the fully-adjusted model was 0.009, consistent with the results when the TyG index is treated as a continuous variable. Additionally, we observed that the trend of the effect size in different TyG index groups was not equidistant (Table 3).

This study analyzed whether there is a linear relationship between the TyG index and Helicobacter pylori infection. After adjusting for covariates, the smooth curve and the result of the generalized additive model indicated a linear relationship between the TyG index and Helicobacter pylori. Both binary logistic regression and two-piecewise binary logistic regression were used to fit the association, and the best-fit model was selected based on the P-value for the log likelihood ratio test. Since the P-value for the log-likelihood ratio test was more than 0.05, the two-piecewise binary logistic regression was chosen to accurately represent the relationship between the TyG index and Helicobacter pylori.

### Subgroup analysis

**Fig 2:**
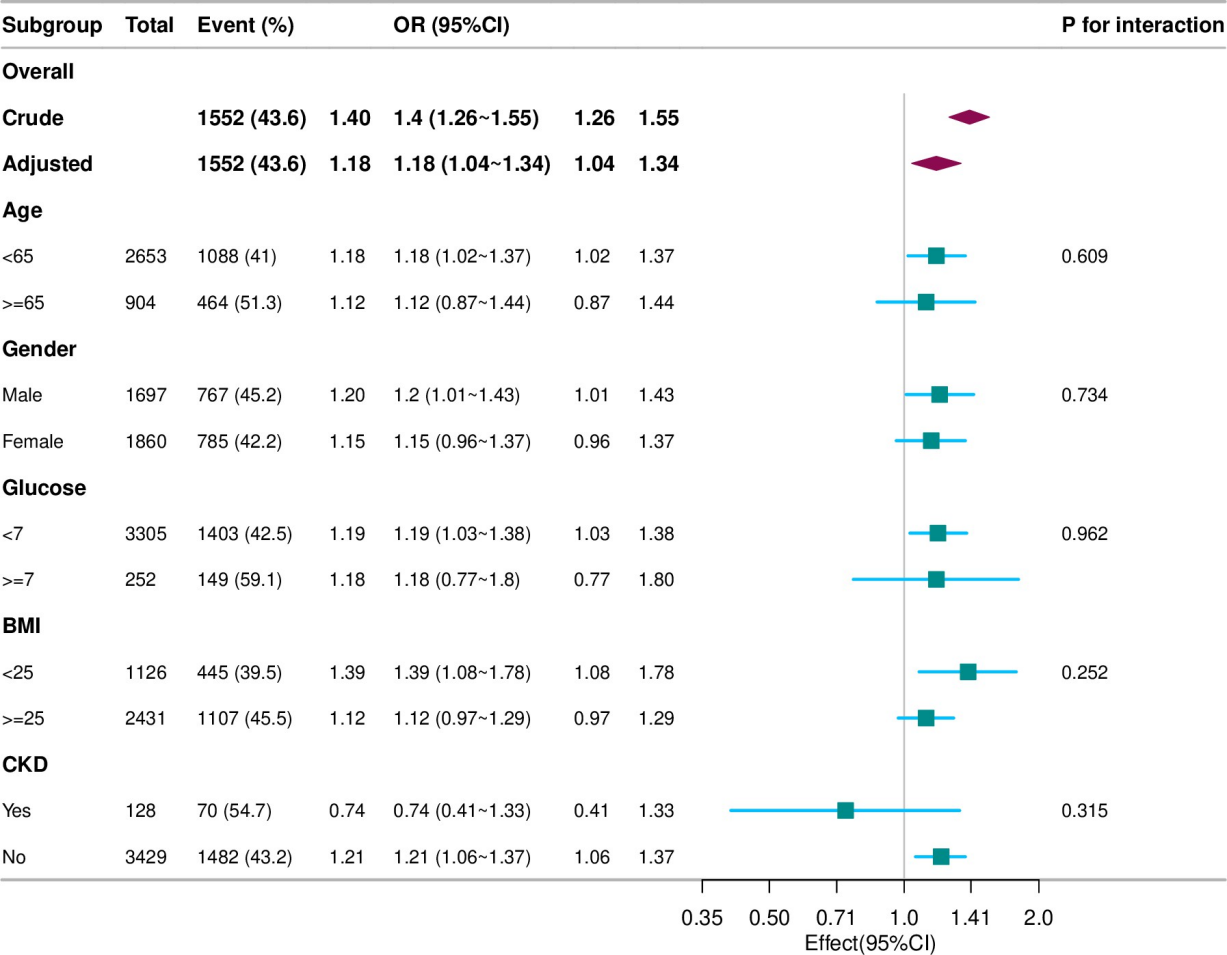
Forest plot of the association of demographic and clinical factors with helicobacter pylori infection in different subgroups.

To observe the trend of effect sizes in different variables, we stratified the analysis by age, sex, glucose (glu), body mass index (bmi), and chronic kidney disease (CKD) (Fig 2). We found that, according to our a priori specifications, no interactions were observed, and the p-values for all interactions were greater than 0.05, indicating robust results.

## Discussion

Our study reveals a significant linear association between the Triglyceride-glucose (TyG) index and Helicobacter pylori (H. pylori) infection, contributing to the understanding of metabolic effects on infectious diseases. The relationship between the TyG index and H. pylori infection is influenced by age, gender, blood glucose levels, BMI, and chronic kidney disease (CKD), without any subgroup interactions, indicating robust results.

These findings align with previous research suggesting that metabolic variables can differently impact infectious diseases. The interaction between H. pylori infection and metabolic health, particularly the role of insulin resistance, is highlighted. Previous studies have indicated that H. pylori infection is an independent risk factor for increased fasting plasma glucose (FPG) levels in non-diabetic individuals, and that H. pylori eradication can improve glycemic control in patients with type 2 diabetes (T2DM) [33–35]. Additionally, H. pylori infection has been associated with increased accumulation of advanced glycation end products (AGEs) in the skin of patients with type 1 diabetes, suggesting a complex interplay between infection and metabolic health [36].

The TyG index, as a marker of insulin resistance, may influence the immune response to H. pylori, potentially affecting bacterial colonization or the host’s susceptibility to infection-related complications. Our study underscores the need for personalized approaches in managing H. pylori-infected patients, as the association between the TyG index and H. pylori infection varies with metabolic health.

The clinical significance of our findings is substantial. Understanding the linear relationship between the TyG index and H. pylori infection could aid clinicians in identifying high-risk individuals for infection or related complications, leading to earlier interventions and tailored treatment strategies. Our study’s larger sample size compared to previous studies enhances the statistical power and generalizability of our findings. We addressed the nonlinearity between variables, providing a more accurate representation of the relationship, and employed strict statistical adjustment methods to minimize the impact of potential confounders, enhancing the internal validity of our results.

However, our study has limitations. The generalizability of our findings is restricted to the NHANES 1999–2000 cycle, potentially limiting the applicability to broader populations. The exclusion of participants with missing data for H. pylori serology and covariates may limit the applicability to these groups. The cross-sectional design precludes the establishment of causality, and the lack of longitudinal data limits our ability to determine the directionality of the observed associations. The mechanisms by which H. pylori affects glycemic control are not yet fully understood and may require further biological experimentation. Future longitudinal studies are necessary to confirm our findings and explore the causal pathways involved.

In conclusion, our study reveals a significant linear relationship between the TyG index and H. pylori infection, influenced by metabolic and demographic factors. This finding underscores the potential of the TyG index as a screening tool in routine health assessments for identifying individuals at risk of H. pylori infection. Future research should investigate the causal mechanisms and explore interventions targeting the TyG index to reduce infection risk, potentially leading to improved patient management strategies.
